# Supplementary material for: Health effects of desert dust and sand storms: a systematic review and meta-analysis protocol
Source: BMJ Open. 2019 Jul 30;9(7):e029876. doi: 10.1136/bmjopen-2019-029876 (PMC6677997; doi:10.1136/bmjopen-2019-029876)
Supplement: Supplementary data [file bmjopen-2019-029876supp002.pdf]

Supplementary File 2.

PubMed search strategy:

(Desert dust[Title/Abstract] OR Sand storms[Title/Abstract] OR Dust episode[Title/Abstract] OR Dust outbreaks[Title/Abstract] OR Asian dust[Title/Abstract] OR Saharan dust[Title/Abstract] OR Yellow dust[Title/Abstract] OR Kosa[Title/Abstract] OR Calima[Title/Abstract]) AND ((mortality[Title/Abstract] OR death[Title/Abstract]) OR (morbidity[Title/Abstract] OR hospital admissions[Title/Abstract] OR emergency admissions[Title/Abstract] OR emergency room[Title/Abstract] OR hospital clinic[Title/Abstract] OR visits[Title/Abstract] OR calls[Title/Abstract]) OR (cardiovascular[Title/Abstract] OR cardicac[Title/Abstract] OR respiratory[Title/Abstract] OR infectious[Title/Abstract] OR coccidiomycosis[Title/Abstract] OR meningitis[Title/Abstract] OR dermatological [Title/Abstract] OR accidents[Title/Abstract])))

EMBASE search strategy:

('Desert dust':ab,ti OR 'Sand storms':ab,ti OR 'Dust episode':ab,ti OR 'Dust outbreaks':ab,ti OR 'Asian dust':ab,ti OR 'Saharan dust':ab,ti OR 'Yellow dust':ab,ti OR 'Kosa':ab,ti OR 'Calima':ab,ti) AND (('mortality':ab,ti OR 'death':ab,ti) OR ('morbidity':ab,ti OR 'hospital admissions':ab,ti OR 'emergency admissions':ab,ti OR 'emergency room':ab,ti OR 'clinic':ab,ti OR 'visits':ab,ti OR 'calls':ab,ti) OR ('cardiovascular':ab,ti OR 'cardicac':ab,ti OR 'respiratory':ab,ti OR 'infectious':ab,ti OR 'coccidiomycosis':ab,ti OR 'meningitis':ab,ti OR 'dermatological':ab,ti OR 'accidents':ab,ti)))
