# Supplementary material for: Health effects of desert dust and sand storms: a systematic review and meta-analysis protocol
Source: BMJ Open. 2019 Jul 30;9(7):e029876. doi: 10.1136/bmjopen-2019-029876 (PMC6677997; doi:10.1136/bmjopen-2019-029876)
Supplement: Author's manuscript [file bmjopen-2019-029876.draft_revisions.pdf]

BMJ Open is committed to open peer review. As part of this commitment we make the peer review history of every article we publish publicly available.

When an article is published we post the peer reviewers' comments and the authors' responses online. We also post the versions of the paper that were used during peer review. These are the versions that the peer review comments apply to.

The versions of the paper that follow are the versions that were submitted during the peer review process. They are not the versions of record or the final published versions. They should not be cited or distributed as the published version of this manuscript.

BMJ Open is an open access journal and the full, final, typeset and author-corrected version of record of the manuscript is available on our site with no access controls, subscription charges or pay-per-view fees (http://bmjopen.bmj.com).

If you have any questions on BMJ Open's open peer review process please email info.bmjopen@bmj.com

BMJ Open

Health effects of desert dust and sand storms: a systematic review and meta-analysis protocol

Journal:	*BMJ Open*
Manuscript ID	bmjopen-2019-029876
Article Type:	Protocol
Date Submitted by the Author:	15-Feb-2019
Complete List of Authors:	Tobias, Aurelio; Spanish National Research Council, Institute of Environmental Assessment and Water Research<br>Karanasiou, Angeliki; Spanish National Research Council, Institute of Environmental Assessment and Water Research<br>Amato, Fulvio; National Spanish Research Council, Institute of Environmental Assessment and Water Research<br>Querol, Xavier; National Spanish Research Council, Institute of Environmental Assessment and Water Research
Keywords:	EPIDEMIOLOGY, PLASTIC & RECONSTRUCTIVE SURGERY, PUBLIC HEALTH

SCHOLARONE™
Manuscripts

Health effects of desert dust and sand storms: a systematic review and meta-analysis protocol

Aurelio Tobías[1,2], Angeliki Karanasiou[1], Fulvio Amato[1], Xavier Querol[1]

1. Institute of Environmental Assessment and Water Research (IDAEA), Spanish Council for Scientific Research (CSIC), Barcelona, Spain.
2. Japan Society for the Promotion of Science (JSPS) Research Fellow.

Address for correspondence:

Dr. Aurelio Tobías

Institute of Environmental Assessment and Water Research (IDAEA),

Spanish Council for Scientific Research (CSIC),

C/ Jordi Girona 18-26

08034 Barcelona, Spain.

e-mail: aurelio.tobias@idaea.csic.es

Telf.: +34 934006100

For peer review only

ABSTRACT

Introduction: Desert dust concentrations raise concerns about adverse effects on human health. During last decade special attention has been given to mineral dust particles from desert dust and sand storms. However, evidence from previous reviews reported inconclusive results on their health effects and biological mechanism still remains unclear. We aim to systematically synthesise evidence on the health effects of desert dust and sand storms accounting for the relevant desert dust patterns from source areas and emissions, transport and composition.

Methods an Analysis: We will conduct a systematic review that investigated the health effects of desert dust and sand storms in any population. The search will be performed for any eligible studies from previous reviews and selected electronic databases from 1999 until 2017. Study selection and reporting will follow the Preferred Reporting Items for Systematic Reviews and Meta-Analyses guidelines. Data from individual studies will be extracted using a standardised data extraction form. Quality of the studies will be assessed using a risk of bias tool for environmental exposures developed by experts convened by the World Health Organisation (WHO). Meta-analysis will be performed by calculating the appropriate effect measures of association for binary and continuous outcomes from individual studies. Subgroup analyses will be performed by geographical areas to account for desert dust patterns.

Ethics and Dissemination: No primary data will be collected thus no formal ethical approval is required. This systematic review will help to fill the research gaps in the knowledge of desert dust on human health. The results will be disseminated though a WHO peer-reviewed publication and conference presentation.

PROSPERO registration number: CRD42018091809

For peer review only

Strengths and limitations of this study

- Desert dust and sand storms represent a serious hazard for the environment and human health.
- The review will systematically retrieve and document all published studies in accordance to a standardised protocol for data collection and reporting of findings.
- Meta-analysis will be used to provide evidence and increase the precision of the quantitative estimates of exposures related to desert dust.
- Subgroup analysis will be performed by geographical area to account for relevant desert dust patterns from source areas, emissions, transport and composition.
- The results will provide evidence to fill the gap in the knowledge of health effects of desert dust and may help to develop appropriate preventive measures for dust episodes.

For peer review only

For peer review only - http://bmjopen.bmj.com/site/about/guidelines.xhtml

INTRODUCTION

Background

Desert dust and sand storms play a significant role in different aspects of weather, climate and atmospheric chemistry and represent a serious hazard for the environment and human health (1,2). Desert dust has a large impact on air quality, not only in areas close to the source points or regions but over areas even a few thousands of kilometres distant (3,4). The air quality influence of dust episodes is a complex issue, since are not only increasing particulate matter ambient concentrations by supplying large loads of mineral (or crustal) dust, but it can also carry anthropogenic pollutants, previously deposited in the source areas or trapped by the high dust air mass during its atmospheric transport (5,6), and can also carry large amounts of microorganisms and toxic biogenic allergens (7,8).

Research on the health effects of desert dust has increased substantially over the last decade. Although epidemiological studies from affected areas suggest the potential health effects of desert dust, the evidence still remains unclear, as well as the biological mechanism. Previous published reviews, systematic or not, reported inconsistent results across studies and geographical regions (9-12). However, the reviewed studies differed in terms of study settings, dust exposure assessment methods and epidemiological study designs. Consequently, none of these reviews allowed to summarizing the evidence quantitatively with meta-analysis. Thus, a systematic review with a proper standardized protocol is required to provide a better knowledge of health effects and mechanism of toxicity of desert dust.

Objective

The objective our systematic review is to summarize the evidence on the health effects of desert dust and sand storms, properly accounting for the relevant desert dust patterns from source areas and emissions, transport and composition. This systematic review will be carried out in the framework of the World Health Organisation (WHO) to inform the update of the WHO Global Air Quality Guidelines (AQGs), coordinated by the WHO Regional Office's European Centre for Environment and Health.

For peer review only

METHODS

This protocol has been registered in PROSPERO (CRD42018091809) and follows the Preferred Reporting Items for Systematic Review and Meta-Analysis Protocols (PRISMA-P) checklist (13) (online supplementary file 1). Any amendments to the protocol will be tracked and dated in PROSPERO.

Study eligibility criteria

The research question considered in this systematic review was established by the WHO at the 3rd Meeting of the Global Platform on Air Quality and Health (Madrid, Spain, March 2017).

Population

This systematic review will include any human population, from developed and developing countries, living in urban and in rural areas exposed to desert dust and sand storms. Vulnerable population subgroups to the effects of desert dust ands sand storms will be included, such as these with specific pre-existing health conditions (e.g. respiratory or cardiovascular diseases), pregnant women, new-borns, children or the elderly. Whenever applicable, the considered health effect of exposure to desert dust in these vulnerable sub-groups of the population will be assessed separately.

Types of exposures

We will consider short and long-term exposure to desert dust characterized by source apportionment, component analysis, particle size and composition, use of weather data and back trajectories, or any other suitable method. The exposure to desert dust should be compared with non-exposure to desert dust or exposure to lower concentrations of desert dust.

For peer review only

Types of outcome measures

We will consider any independent measurable change in the risk of adverse health effects (incidence and/or prevalence) related to exposure to desert dust. Considering an evaluation of the human health disorders related to desert dust (14), we will consider the following outcomes in this systematic review: mortality for all-natural causes and cause-specific (e.g., cardiovascular and respiratory); hospital admissions, emergency room admissions and emergency department specific causes; respiratory and cardiovascular symptoms; coccidiomycosis; meningococcal meningitis; dermatological disorder; and deaths or injuries resulting from transport accidents.

Types of studies

This systematic review will consider all types of relevant studies published in peer-reviewed journals that assess for the health effects of desert dust and sand storms. However, the studies will be classifying according to their observational or experimental design (e.g., quasi-experimental and randomised controlled trials). The observational studies will also be classifying at the ecological or individual level (e.g., surveys, case-control, cohort studies).

Information sources

We will systematically conduct a comprehensive literature search. Firstly, we will identify all studies already reported in previous reviews, systematic or not, on the health effects of desert dust and sand storms (9-12). Next, we will conduct a literature search for studies matching the study eligibility criteria in various databases, including MEDLINE (using PubMed) and EMBASE. Finally, references of identified relevant studies will be scanned to identify additional published data matching the study eligibility criteria. The search strategy will be developed using free-text for each search engine, considering different definitions and keywords for desert dust and sand storms and health outcomes. We will retrieve eligible published studies from 1999 to 2017 without language restrictions and will translate the non-English abstracts and studies, if necessary.

For peer review only

Selection of studies

Two reviewers will screen the titles and abstracts independently, record and import search results of their findings in EndNote X7 (Clarivate Analytics). All studies will be classified for inclusion or exclusion based on the eligibility of this systematic review and further checked for duplication. Full-text article will be retrieved if the study is unclear from the title and/or abstract and the relevant studies will be further confirmed for inclusion through full-text review. The third reviewer will resolve discrepancies between reviewers.

Data extraction

Reviewers will use a standardised data extraction form using Excel spreadsheet (Microsoft Corporation). Here, if a given article reports several health outcomes of interest, each outcome will take one record. Likewise, if a given article reports age and/or gender-specific effects, these will also be collected. Moreover, if a given study reports more than one effect estimate for each health outcome (e.g., for several lags) the one the authors favoured most will be extracted. Main conclusion/findings for each study will also be collected as free text. The following characteristics of the included studies will be extracted:

- Citation details (e.g., first author, title, journal and date of publication).
- Study location (e.g. city, country, region), study period, characteristics of the study population (e.g., age, gender).
- Study design and statistical analysis.
- Method to identify dust events and/or exposure levels for days with and without dust events.
- Outcome assessment (e.g., cause of mortality, morbidity and other cause-specific outcomes).
- Confounders measured and adjusted for (e.g., meteorological variables and other air pollutants).
- Effect measures of association, and its 95% confidence interval, for days with and without dust events.

For peer review only

Quality assessment

A new domain-based risk of bias tool, currently under development by a group of experts on environmental exposures convened by the WHO Guideline Development Group (GDG) for the AQGs, will be used for the assessment whenever it becomes available. The risk of bias tool will be revised and adapted to by the systematic review team members. Two reviewers will assess the risk of bias independently and disagreements will be resolved through discussion. A member of the WHO group of experts will support the application of the tool.

Data synthesis and statistical analysis

For binary outcomes, the relative risk (RR) will be used as the common effect measure of association across studies. The hazard ratio (HR) if reported, may be considered equivalent to RRs. If Odds Ratios (OR) are reported in the study and the outcome prevalence is higher than 10%, or the study design allows for it, they will be recalculated as RRs. Meta-analysis input data will be as the percentage increase in the RR (e.g., as %RR = (RR-1) x100%). When reporting effects of particulate matter for days with and without dust events the %RR will be standardised for a given increment of 10 μg/m^3, assuming a linear exposure-outcome relationship. For continuous outcomes, effect measures such as weighted mean difference (WMD) or standardized mean difference (SMD) may be used. The scale of the available data will be primarily used to determine the choice of effect measure if studies report effect estimates with the same or similar scale (WMD) or when the outcome is measured using different scales (SMD) (15).

Effect measures of association from individual studies will be summarized in a meta-analysis estimate, with its 95% confidence interval, based on fixed or random effects model, depending on the heterogeneity of the analysis (15,16). Statistical heterogeneity of the effect estimates between studies included in the meta-analysis will be assessed using the I^2 statistic, where values of 25%, 50% and 75% will be considered as low, moderate and high degree of heterogeneity, respectively (17). We will also report the between-study variance

(τ^2) and the Cochran Q test for heterogeneity (18). All analyses will be done using Stata Statistical Software, release 15 (Stata Corp LP)

Due to the relevance of the study setting and dust exposure assessment, subgroup analyses will be done by geographical areas, considering studies conducted in Eastern Asia, Europe, Middle the East and other regions (e.g., Australia and North America). Subgroup analyses by age groups (14, 15-65, 65 years) and gender (male and female) will be also considered whenever data become available in the individual studies.

Assessment of reporting biases

Publication bias will be assessed when at least 10 studies be included in the meta-analysis, using Begg's rank correlation test and examined using funnel plots (19).

Reporting conclusions

The systematic review will be conducted and reported in accordance to the Preferred Reporting Items for Systematic Review and Meta-Analysis (PRISMA) (20) standards, with slight adaptations since these were originally intended for health care intervention evaluation. We will base our conclusions only on findings from the quantitative or narrative synthesis of included studies for this systematic review. The strength of the evidence will be assessed using an adaptation of the Grading of Recommendations Assessment (GRADE) (21), undergo by the working group on GRADE adaptation for environmental studies convened by the GDG for the AQGs. The conclusions from this systematic review will be used to inform the update of the WHO AQGs, coordinated by the WHO Regional Office's European Centre for Environment and Health.

Ethics and dissemination

Formal ethical approval is not required, as no primary data will be collected. The results of this systematic will fill the current research gaps in the knowledge on the health effects of desert dust and sand storms and would also help to alert the most sensitive population

For peer review only

taking appropriate measures during during dust episodes. All findings will be shared and disseminated by WHO in a peer-reviewed publication and conference presentation.

Contributors: AT is the guarantor. AT drafted the protocol and registered the protocol in PROSPERO. XQ reviewed and commented on the protocol in PROSPERO. AK, FA and XQ all reviewed and commented on this protocol.

Funding: The World Health Organization, supported by the Ministry of Foreign Affairs of Norway, has funded this systematic review.

Competing interests: None declared.

Provenance and peer review: Commissioned by WHO; externally peer reviewed.

For peer review only

References

1. Mahowald NM, Kloster S, Engelstaedter S, Moore JK, Mukhopadhyay S, McConnell JR, et al. Observed 20th century desert dust variability: impact on climate and biogeochemistry. Atmospheric Chemistry and Physics. 2010;10(22):10875-93.

2. De Longueville F, Hountondji YC, Henry S, Ozer P. What do we know about effects of desert dust on air quality and human health in West Africa compared to other regions? Sci Total Environ. 2010;409(1):1-8.

3. Prospero JM. Environmental characterization of global sources of atmospheric soil dust identified with the NIMBUS 7 Total Ozone Mapping Spectrometer (TOMS) absorbing aerosol product. Reviews of Geophysics. 2002;40(1):1002.

4. Ginoux P, Prospero JM, Gill TE, Hsu NC, Zhao M. Global-scale attribution of anthropogenic and natural dust sources and their emission rates based on MODIS Deep Blue aerosol products. Reviews of Geophysics. 2012;50(3):1-36.

5. Mori I. Change in size distribution and chemical composition of kosa (Asian dust) aerosol during long-range transport. Atmospheric Environment. 2003;37(30):4253-63.

6. Rodríguez S, Alastuey A, Alonso-Pérez S, Querol X, Cuevas E, Abreu-Afonso J, et al. Transport of desert dust mixed with North African industrial pollutants in the subtropical Saharan Air Layer. Atmospheric Chemistry and Physics. 2011;11(13):6663-85.

7. Ho H-M, Rao CY, Hsu H-H, Chiu Y-H, Liu C-M, Chao HJ. Characteristics and determinants of ambient fungal spores in Hualien, Taiwan. Atmospheric Environment. 2005;39(32):5839-50.

8. Griffin DW, Garrison VH, Herman JR, Shinn EA. African desert dust in the Caribbean atmosphere: Microbiology and public health. Aerobiologia. 2001;17(3):203-13.

9. Hashizume M, Ueda K, Nishiwaki Y, Michikawa T, Onozuka D. Health effects of Asian dust events: a review of the literature. Nihon Eiseigaku Zasshi. 2010;65(3):413-21.

10. Karanasiou A, Moreno N, Moreno T, Viana M, de Leeuw F, Querol X. Health effects from Sahara dust episodes in Europe: Literature review and research gaps. Environment International. 2012;47:107-14.

For peer review only - http://bmjopen.bmj.com/site/about/guidelines.xhtml

11. De Longueville F, Ozer P, Doumbia S, Henry S. Desert dust impacts on human health: an alarming worldwide reality and a need for studies in West Africa. Int J Biometeorol. 2013;57(1):1-19.

12. Zhang X, Zhao L, Tong D, Wu G, Dan M, Teng B. A Systematic Review of Global Desert Dust and Associated Human Health Effects. Atmosphere. 2016;7(12).

13. Shamseer L, Moher D, Clarke M, Ghersi D, Liberati A, Petticrew M, et al. Preferred reporting items for systematic review and meta-analysis protocols (PRISMA-P) 2015: elaboration and explanation. BMJ. 2015;349(jan02 1):g7647-g.

14. Goudie AS. Desert dust and human health disorders. Environment International. 2014;63:101-13.

15. Higgins JPT, Green S (editors). Cochrane handbook for systematic reviews of interventions version 5.1.0 [updated March 2011]. The Cochrane Collaboration; 2011. Available from http://www.handbook.cochrane.org. (accessed 15.02.2019).

16. Borenstein M, Hedges LV, Higgins JPT, Rothstein HR. A basic introduction to fixed-effect and random-effects models for meta-analysis. Research Synthesis Methods. 2010;1(2):97-111.

17. Higgins JP, Thompson SG. Quantifying heterogeneity in a meta-analysis. Stat Med. 2002;21(11):1539-58.

18. Cochran WG. The χ test of goodness of fit. Annals of Mathematical Statistics 1952;23:315-45.

19. Begg CB, Mazumdar M. Operating characteristics of a rank correlation test for publication bias. Biometrics. 1994;50(2):1088-101.

20. Moher D, Liberati A, Tetzlaff J, Altman DG. Preferred reporting items for systematic reviews and meta-analyses: the PRISMA statement. BMJ. 2009;339(jul21 1):b2535-b.

21. Guyatt GH, Oxman AD, Vist GE, et al. GRADE Working Group. GRADE: an emerging consensus on rating quality of evidence and strength of recommendations. BMJ. 2008;336:924-6.

For peer review only

PRISMA-P (Preferred Reporting Items for Systematic review and Meta-Analysis Protocols) 2015 checklist: recommended items to address in a systematic review protocol*

Section and topic	Item No.	Checklist item	
**ADMINISTRATIVE INFORMATION**			
Title:			
Identification	1a	Identify the report as a protocol of a systematic review	Page 1
Update	1b	If the protocol is for an update of a previous systematic review, identify as such	N/A
Registration	2	If registered, provide the name of the registry (such as PROSPERO) and registration number	Pages 2-5
Authors:			
Contact	3a	Provide name, institutional affiliation, e-mail address of all protocol authors; provide physical mailing address of corresponding author	Page 1
Contributions	3b	Describe contributions of protocol authors and identify the guarantor of the review	Page 10
Amendments	4	If the protocol represents an amendment of a previously completed or published protocol, identify as such and list changes; otherwise, state plan for documenting important protocol amendments	Page 5
Support:			
Sources	5a	Indicate sources of financial or other support for the review	Page 10
Sponsor	5b	Provide name for the review funder and/or sponsor	Page 10

For peer review only

Role of sponsor or funder	5c	Describe roles of funder(s), sponsor(s), and/or institution(s), if any, in developing the protocol	Page 10
INTRODUCTION			
Rationale	6	Describe the rationale for the review in the context of what is already known	Page 4
Objectives	7	Provide an explicit statement of the question(s) the review will address with reference to participants, interventions, comparators, and outcomes (PICO)	Page 4-6
METHODS			
Eligibility criteria	8	Specify the study characteristics (such as PICO, study design, setting, time frame) and report characteristics (such as years considered, language, publication status) to be used as criteria for eligibility for the review	Pages 5-6
Information sources	9	Describe all intended information sources (such as electronic databases, contact with study authors, trial registers or other grey literature sources) with planned dates of coverage	Page 6
Search strategy	10	Present draft of search strategy to be used for at least one electronic database, including planned limits, such that it could be repeated	Page 6
Study records:			
Data management	11a	Describe the mechanism(s) that will be used to manage records and data throughout the review	Page 7
Selection process	11b	State the process that will be used for selecting studies (such as two independent reviewers) through each phase of the review (that is, screening, eligibility and inclusion in meta-analysis)	Pages 7-8

For peer review only

Data collection process	11c	Describe planned method of extracting data from reports (such as piloting forms, done independently, in duplicate), any processes for obtaining and confirming data from investigators	Page 7
Data items	12	List and define all variables for which data will be sought (such as PICO items, funding sources), any pre-planned data assumptions and simplifications	Pages 7
Outcomes and prioritization	13	List and define all outcomes for which data will be sought, including prioritization of main and additional outcomes, with rationale	Pages 6
Risk of bias in individual studies	14	Describe anticipated methods for assessing risk of bias of individual studies, including whether this will be done at the outcome or study level, or both; state how this information will be used in data synthesis	Page 8
Data synthesis	15a	Describe criteria under which study data will be quantitatively synthesised	Pages 8-9
	15b	If data are appropriate for quantitative synthesis, describe planned summary measures, methods of handling data and methods of combining data from studies, including any planned exploration ofc onsistency (such as I^2, Kendall's τ)	Pages 8-9
	15c	Describe any proposed additional analyses (such as sensitivity or subgroup analyses, meta-regression)	Pages 8-9
	15d	If quantitative synthesis is not appropriate, describe the type of summary planned	N/A
Meta-bias(es)	16	Specify any planned assessment of meta-bias(es) (such as publication bias across studies, selective reporting within studies)	Page 9
Confidence in cumulative evidence	17	Describe how the strength of the body of evidence will be assessed (such as GRADE)	Page 9

For peer review only

*** It is strongly recommended that this checklist be read in conjunction with the PRISMA-P Explanation and Elaboration (cite when available) for important clarification on the items. Amendments to a review protocol should be tracked and dated. The copyright for PRISMA-P (including checklist) is held by the PRISMA-P Group and is distributed under a Creative Commons Attribution Licence 4.0.**

From: Shamseer L, Moher D, Clarke M, Ghersi D, Liberati A, Petticrew M, Shekelle P, Stewart L, PRISMA-P Group. Preferred reporting items for systematic review and meta-analysis protocols (PRISMA-P) 2015: elaboration and explanation. BMJ. 2015;349(jan02 1):g7647.

For peer review only

BMJ Open

Health effects of desert dust and sand storms: a systematic review and meta-analysis protocol

Journal:	*BMJ Open*
Manuscript ID	bmjopen-2019-029876.R1
Article Type:	Protocol
Date Submitted by the Author:	01-May-2019
Complete List of Authors:	Tobias, Aurelio; Spanish National Research Council, Institute of Environmental Assessment and Water Research<br>Karanasiou, Angeliki; Spanish National Research Council, Institute of Environmental Assessment and Water Research<br>Amato, Fulvio; National Spanish Research Council, Institute of Environmental Assessment and Water Research<br>Querol, Xavier; National Spanish Research Council, Institute of Environmental Assessment and Water Research
**Primary Subject Heading**:	Occupational and environmental medicine
Secondary Subject Heading:	Global health, Public health, Epidemiology
Keywords:	EPIDEMIOLOGY, PUBLIC HEALTH, Health policy < HEALTH SERVICES ADMINISTRATION & MANAGEMENT

SCHOLARONE™
Manuscripts

Health effects of desert dust and sand storms: a systematic review and meta-analysis protocol

Aurelio Tobías[1], Angeliki Karanasiou[1], Fulvio Amato[1], Xavier Querol[1]

1. Institute of Environmental Assessment and Water Research (IDAEA), Spanish Council for Scientific Research (CSIC), Barcelona, Spain.

Address for correspondence:

Dr Aurelio Tobías

Institute of Environmental Assessment and Water Research (IDAEA),

Spanish Council for Scientific Research (CSIC),

C/ Jordi Girona 18-26

08034 Barcelona, Spain.

e-mail: aurelio.tobias@idaea.csic.es

Telf.: +34 934006100

For peer review only

ABSTRACT

Introduction: Desert dust concentrations raise concerns about adverse effects on human health. During the last decade, special attention has been given to mineral dust particles from desert dust and sand storms. However, evidence from previous reviews reported inconclusive results on their health effects and biological mechanism still remains unclear. We aim to systematically synthesise evidence on the health effects of desert dust and sand storms accounting for the relevant desert dust patterns from source areas and emissions, transport and composition.

Methods an Analysis: We will conduct a systematic review that investigated the health effects of desert dust and sand storms in any population. The search will be performed for any eligible studies from previous reviews and selected electronic databases until 2018. Study selection and reporting will follow the Preferred Reporting Items for Systematic Reviews and Meta-Analyses guidelines. Data from individual studies will be extracted using a standardised data extraction form. Quality of the studies will be assessed using a risk of bias tool for environmental exposures developed by experts convened by the World Health Organisation (WHO). Meta-analysis will be performed by calculating the appropriate effect measures of association for binary and continuous outcomes from individual studies. Subgroup analyses will be performed by geographical areas to account for desert dust patterns.

Ethics and Dissemination: No primary data will be collected thus no formal ethical approval is required. This systematic review will help to fill the research gaps in the knowledge of desert dust on human health. The results will be disseminated through a WHO peer-reviewed publication and a conference presentation.

PROSPERO registration number: CRD42018091809

For peer review only

Strengths and limitations of this study

- This will be the first systematic review to retrieve published studies on the health effects of desert dust in accordance with a standardised protocol for data collection and reporting of findings.
- Meta-analysis will be used to provide evidence of the quantitative estimates of the health effects of desert dust exposures, accounting for relevant desert dust patterns from source areas, emissions, transport and composition.
- The diversity of methods to identify dust exposure events and the extensive health effects attributed to desert dust exposures might lead to study designs with different methodological characteristics, which should be carefully considered.
- The results of this systematic review will provide evidence to fill the gap in the knowledge of the health effects of desert dust and may help to develop appropriate preventive measures for dust episodes.

For peer review only

INTRODUCTION

Background

Desert dust and sand storms play a significant role in different aspects of weather, climate and atmospheric chemistry and represent a serious hazard for the environment and human health (1,2). Desert dust has a large impact on air quality, not only in areas close to the source points or regions but over areas even a few thousands of kilometres distant (3,4). The air quality influence of dust episodes is a complex issue, since are not only increasing particulate matter ambient concentrations by supplying large loads of mineral (or crustal) dust, but it can also carry anthropogenic pollutants, previously deposited in the source areas or trapped by the high dust air mass during its atmospheric transport (5,6), and can also carry large amounts of microorganisms and toxic biogenic allergens (7,8).

Research on the health effects of desert dust has increased substantially over the last decade. Although epidemiological studies from affected areas suggest the potential health effects of desert dust, the evidence still remains unclear, as well as the biological mechanism. Previously published reviews, systematic or not, reported inconsistent results across studies and geographical regions (9-12). However, the reviewed studies differed in terms of study settings, dust exposure assessment methods and epidemiological study designs. Consequently, none of these reviews allowed to summarizing the evidence quantitatively with meta-analysis. Thus, a systematic review with a proper standardized protocol is required to provide a better knowledge of health effects and mechanism of toxicity of desert dust.

Objective

The objective of our systematic review is to summarize the evidence on the health effects of desert dust and sand storms, properly accounting for the relevant desert dust patterns from source areas and emissions, transport and composition. This systematic review will be carried out in the framework of the World Health Organisation (WHO) to inform the update of the WHO Global Air Quality Guidelines (AQGs), coordinated by the WHO Regional Office's European Centre for Environment and Health.

For peer review only - http://bmjopen.bmj.com/site/about/guidelines.xhtml

For peer review only

METHODS

This protocol has been registered in PROSPERO (CRD42018091809) and follows the Preferred Reporting Items for Systematic Review and Meta-Analysis Protocols (PRISMA-P) checklist (13) (online supplementary file 1). Any amendments to the protocol will be tracked and dated in PROSPERO.

Patient and public involvement

Patients and or public will not be involved in this study as this is a systematic review protocol.

Study eligibility criteria

The research question considered in this systematic review was established by the WHO at the 3rd Meeting of the Global Platform on Air Quality and Health (Madrid, Spain, March 2017).

Population

This systematic review will include any human population, from developed and developing countries, living in urban and in rural areas exposed to desert dust and sand storms. Vulnerable population subgroups to the effects of desert dust and sand storms will be included, such as these with specific pre-existing health conditions (e.g. respiratory or cardiovascular diseases), pregnant women, newborns, children or the elderly. Whenever applicable, the considered health effect of exposure to desert dust in these vulnerable sub-groups of the population will be assessed separately.

Types of exposures

We will consider short and long-term exposure to desert dust characterized by source apportionment, component analysis, particle size and composition, use of weather data and

For peer review only

back trajectories, or any other suitable method. The exposure to desert dust should be compared with non-exposure to desert dust or exposure to lower concentrations of desert dust.

Types of outcome measures

We will consider any independent measurable change in the risk of adverse health effects (incidence and/or prevalence) related to exposure to desert dust as mortality for all-natural causes and cause-specific (e.g., cardiovascular and respiratory) and hospital admissions, emergency room admissions and emergency department specific causes. Moreover, based on a previous evaluation of the human health disorders related to desert dust (14), we will also consider cardiovascular and respiratory symptoms; lung diseases; coccidiomycosis; meningococcal meningitis; dermatological disorder; and deaths or injuries resulting from transport accidents.

Types of studies

This systematic review will consider all types of relevant studies published in peer-reviewed journals that assess the health effects of desert dust and sand storms. However, the studies will be classified according to their observational or experimental design (e.g., quasi-experimental and randomised controlled trials). The observational studies will also be classified at the ecological or individual level (e.g., surveys, case-control, cohort studies).

For peer review only

Information sources

We will systematically conduct a comprehensive literature search. Firstly, we will identify all studies already reported in previous reviews, systematic or not, on the health effects of desert dust and sand storms (9-12). Next, we will conduct a literature search for studies matching the study eligibility criteria in various databases, including MEDLINE (using PubMed) and EMBASE. Finally, references of identified relevant studies will be scanned to identify additional published data matching the study eligibility criteria. The search strategy will be developed using free-text for each search engine, considering different definitions and keywords for desert dust and sand storms and health outcomes. We will retrieve

For peer review only - http://bmjopen.bmj.com/site/about/guidelines.xhtml

eligible published studies until 2018 without language restrictions and will translate the non-English abstracts and studies, if necessary.

Selection of studies

Two reviewers will screen the titles and abstracts independently, record and import search results of their findings in EndNote X7 (Clarivate Analytics). All studies will be classified for inclusion or exclusion based on the eligibility of this systematic review and further checked for duplication. Full-text articles will be retrieved if the study is unclear from the title and/or abstract and the relevant studies will be further confirmed for inclusion through full-text review. The third reviewer will resolve discrepancies between reviewers.

Data extraction

Reviewers will use a standardised data extraction form using Excel spreadsheet (Microsoft Corporation). Here, if a given study reports several health outcomes of interest, each outcome will take one record. Likewise, if a given study reports age and/or gender-specific effects, these will also be collected. Moreover, if a given study reports more than one effect estimate for each health outcome (e.g., for several lags) the one the authors favoured most will be extracted. Main conclusion/findings for each study will also be collected as free text. The following characteristics of the included studies will be extracted:

- Citation details (e.g., first author, title, journal and date of publication).
- Study location (e.g. city, country, region), study period, characteristics of the study population (e.g., age, gender).
- Study design and statistical analysis.
- Method to identify dust events and/or exposure levels for days with and without dust events.
- Outcome assessment (e.g., cause of mortality, morbidity and other cause-specific outcomes).

For peer review only

- Confounders measured and adjusted for (e.g., meteorological variables and other air pollutants).
- Effect measures of association, and its 95% confidence interval, for days with and without dust events.

Quality assessment

We will use a new domain-based risk of bias tool to assess the quality of the evidence across studies for associations between specific air-pollutants and adverse health outcomes. The risk of bias tool, currently under development by a group of experts on environmental exposures convened by the WHO Guideline Development Group (GDG) for the AQGs, will adapt the domains according to the needs of the observational studies of exposure that form the body of the evidence in air-pollution, it will be revised and adapted to by the systematic review team members and a member of the WHO GDG will support the application of the tool. Two reviewers will assess the risk of bias independently and disagreements will be resolved through discussion.

Data synthesis and statistical analysis

For binary outcomes (e.g., mortality and morbidity), the relative risk (RR) will be used as the common effect measure of association across studies. The hazard ratio (HR) if reported, may be considered equivalent to RRs. If Odds Ratios (OR) are reported in a given study and the outcome prevalence is higher than 10% they will be approximated as RRs (15) and similarly if the study design allows for it (e.g., time-series and time-stratified case-crossover (16)). Meta-analysis input data will be as the percentage increase in the RR (e.g., as %RR = (RR-1) x100%). When reporting effects of particulate matter for days with and without dust events the %RR will be standardised for a given increment of 10 $\mu g/m^3$, assuming a linear exposure-outcome relationship. For continuous outcomes, effect measures such as weighted mean difference (WMD) or standardized mean difference (SMD) may be used. The scale of the available data will be primarily used to determine the choice of effect measure if studies report effect estimates with the same or similar scale (WMD) or when the outcome is measured using different scales (SMD) (15).

For peer review only - http://bmjopen.bmj.com/site/about/guidelines.xhtml

Effect measures of association from individual studies will be summarized in a meta-analysis estimate, with its 95% confidence interval, based on fixed or random effects model, depending on the heterogeneity of the analysis (15,17). Statistical heterogeneity of the effect estimates between studies included in the meta-analysis will be assessed using the I^2 statistic, where values of 25%, 50% and 75% will be considered as a low, moderate and high degree of heterogeneity, respectively (18). We will also report the between-study variance (τ^2) and the Cochran Q test for heterogeneity (19). All analyses will be done using Stata Statistical Software, release 15 (Stata Corp LP)

Due to the relevance of the study setting and dust exposure assessment, subgroup analyses will be done by geographical areas, considering studies conducted in Eastern Asia, Europe, Middle the East and other regions (e.g., Australia and North America). Subgroup analyses by pre-existing health conditions, age groups (14, 15-64, ≥65 years) and gender (male and female) will be also considered whenever data become available in the individual studies.

Assessment of reporting biases

Publication bias will be assessed when at least 10 studies are included in the meta-analysis, using Begg's rank correlation test and examined using funnel plots (20).

Reporting conclusions

The systematic review will be conducted and reported in accordance to the Preferred Reporting Items for Systematic Review and Meta-Analysis (PRISMA) (21) standards, with slight adaptations since these were originally intended for health care intervention evaluation. We will base our conclusions only on findings from the quantitative or narrative synthesis of included studies for this systematic review. The strength of the evidence will be assessed using an adaptation of the Grading of Recommendations Assessment (GRADE) (22), undergo by the working group on GRADE adaptation for environmental studies convened by the GDG for the AQGs. The conclusions from this systematic review will be

For peer review only

For peer review only - http://bmjopen.bmj.com/site/about/guidelines.xhtml

used to inform the update of the WHO AQGs, coordinated by the WHO Regional Office's European Centre for Environment and Health.

Ethics and dissemination

Formal ethical approval is not required, as no primary data will be collected. The results of this systematic review will fill the current research gaps in the knowledge on the health effects of desert dust and sand storms and would also help to alert the most sensitive population taking appropriate measures during dust episodes. All findings will be shared and disseminated by WHO in a peer-reviewed publication and conference presentation.

Contributors: AT is the guarantor. AT drafted the protocol and registered the protocol in PROSPERO. XQ reviewed and commented on the protocol in PROSPERO. AK, FA and XQ all reviewed and commented on this protocol.

Funding: The World Health Organization, supported by the Ministry of Foreign Affairs of Norway, has funded this systematic review.

Competing interests: None declared.

Provenance and peer review: Commissioned by WHO; externally peer reviewed.

For peer review only

References

1. Mahowald NM, Kloster S, Engelstaedter S, Moore JK, Mukhopadhyay S, McConnell JR, et al. Observed 20th-century desert dust variability: impact on climate and biogeochemistry. Atmospheric Chemistry and Physics. 2010;10(22):10875-93.
2. De Longueville F, Hountondji YC, Henry S, Ozer P. What do we know about the effects of desert dust on air quality and human health in West Africa compared to other regions? Sci Total Environ. 2010;409(1):1-8.
3. Prospero JM. Environmental characterization of global sources of atmospheric soil dust identified with the NIMBUS 7 Total Ozone Mapping Spectrometer (TOMS) absorbing aerosol product. Reviews of Geophysics. 2002;40(1):1002.
4. Ginoux P, Prospero JM, Gill TE, Hsu NC, Zhao M. Global-scale attribution of anthropogenic and natural dust sources and their emission rates based on MODIS Deep Blue aerosol products. Reviews of Geophysics. 2012;50(3):1-36.
5. Mori I. Change in size distribution and chemical composition of Kosa (Asian dust) aerosol during long-range transport. Atmospheric Environment. 2003;37(30):4253-63.
6. Rodríguez S, Alastuey A, Alonso-Pérez S, Querol X, Cuevas E, Abreu-Afonso J, et al. Transport of desert dust mixed with North African industrial pollutants in the subtropical Saharan Air Layer. Atmospheric Chemistry and Physics. 2011;11(13):6663-85.
7. Ho H-M, Rao CY, Hsu H-H, Chiu Y-H, Liu C-M, Chao HJ. Characteristics and determinants of ambient fungal spores in Hualien, Taiwan. Atmospheric Environment. 2005;39(32):5839-50.
8. Griffin DW, Garrison VH, Herman JR, Shinn EA. African desert dust in the Caribbean atmosphere: Microbiology and public health. Aerobiologia. 2001;17(3):203-13.
9. Hashizume M, Ueda K, Nishiwaki Y, Michikawa T, Onozuka D. Health effects of Asian dust events: a review of the literature. Nihon Eiseigaku Zasshi. 2010;65(3):413-21.
10. Karanasiou A, Moreno N, Moreno T, Viana M, de Leeuw F, Querol X. Health effects from Sahara dust episodes in Europe: Literature review and research gaps. Environment International. 2012;47:107-14.

For peer review only

11. De Longueville F, Ozer P, Doumbia S, Henry S. Desert dust impacts on human health: an alarming worldwide reality and a need for studies in West Africa. Int J Biometeorol. 2013;57(1):1-19.

12. Zhang X, Zhao L, Tong D, Wu G, Dan M, Teng B. A Systematic Review of Global Desert Dust and Associated Human Health Effects. Atmosphere. 2016;7(12).

13. Shamseer L, Moher D, Clarke M, Ghersi D, Liberati A, Petticrew M, et al. Preferred reporting items for systematic review and meta-analysis protocols (PRISMA-P) 2015: elaboration and explanation. BMJ. 2015;349(jan02 1):g7647-g.

14. Goudie AS. Desert dust and human health disorders. Environment International. 2014;63:101-13.

15. Higgins JPT, Green S (editors). Cochrane handbook for systematic reviews of interventions version 5.1.0 [updated March 2011]. The Cochrane Collaboration; 2011. Available from http://www.handbook.cochrane.org. (accessed 15.02.2019).

16. Lu Y, Zeger SL. On the equivalence of case-crossover and time series methods in environmental epidemiology. Biostatistics. 2007;8(2): 337-44.

17. Borenstein M, Hedges LV, Higgins JPT, Rothstein HR. A basic introduction to fixed-effect and random-effects models for meta-analysis. Research Synthesis Methods. 2010;1(2):97-111.

18. Higgins JP, Thompson SG. Quantifying heterogeneity in a meta-analysis. Stat Med. 2002;21(11):1539-58.

19. Cochran WG. The χ test of goodness of fit. Annals of Mathematical Statistics 1952;23:315-45.

20. Begg CB, Mazumdar M. Operating characteristics of a rank correlation test for publication bias. Biometrics. 1994;50(2):1088-101.

21. Moher D, Liberati A, Tetzlaff J, Altman DG. Preferred reporting items for systematic reviews and meta-analyses: the PRISMA statement. BMJ. 2009;339(jul21 1):b2535-b.

22. Guyatt GH, Oxman AD, Vist GE, et al. GRADE Working Group. GRADE: an emerging consensus on rating quality of evidence and strength of recommendations. BMJ. 2008;336:924-6.

For peer review only - http://bmjopen.bmj.com/site/about/guidelines.xhtml

PRISMA-P (Preferred Reporting Items for Systematic review and Meta-Analysis Protocols) 2015 checklist: recommended items to address in a systematic review protocol*

Section and topic	Item No.	Checklist item	
**ADMINISTRATIVE INFORMATION**			
Title:			
Identification	1a	Identify the report as a protocol of a systematic review	Page 1
Update	1b	If the protocol is for an update of a previous systematic review, identify as such	N/A
Registration	2	If registered, provide the name of the registry (such as PROSPERO) and registration number	Pages 2-5
Authors:			
Contact	3a	Provide name, institutional affiliation, e-mail address of all protocol authors; provide physical mailing address of corresponding author	Page 1
Contributions	3b	Describe contributions of protocol authors and identify the guarantor of the review	Page 10
Amendments	4	If the protocol represents an amendment of a previously completed or published protocol, identify as such and list changes; otherwise, state plan for documenting important protocol amendments	Page 5
Support:			
Sources	5a	Indicate sources of financial or other support for the review	Page 10
Sponsor	5b	Provide name for the review funder and/or sponsor	Page 10

For peer review only

Role of sponsor or funder	5c	Describe roles of funder(s), sponsor(s), and/or institution(s), if any, in developing the protocol	Page 10
INTRODUCTION			
Rationale	6	Describe the rationale for the review in the context of what is already known	Page 4
Objectives	7	Provide an explicit statement of the question(s) the review will address with reference to participants, interventions, comparators, and outcomes (PICO)	Page 4-6
METHODS			
Eligibility criteria	8	Specify the study characteristics (such as PICO, study design, setting, time frame) and report characteristics (such as years considered, language, publication status) to be used as criteria for eligibility for the review	Pages 5-6
Information sources	9	Describe all intended information sources (such as electronic databases, contact with study authors, trial registers or other grey literature sources) with planned dates of coverage	Page 6
Search strategy	10	Present draft of search strategy to be used for at least one electronic database, including planned limits, such that it could be repeated	Page 6
Study records:			
Data management	11a	Describe the mechanism(s) that will be used to manage records and data throughout the review	Page 7
Selection process	11b	State the process that will be used for selecting studies (such as two independent reviewers) through each phase of the review (that is, screening, eligibility and inclusion in meta-analysis)	Pages 7-8

For peer review only

Data collection process	11c	Describe planned method of extracting data from reports (such as piloting forms, done independently, in duplicate), any processes for obtaining and confirming data from investigators	Page 7
Data items	12	List and define all variables for which data will be sought (such as PICO items, funding sources), any pre-planned data assumptions and simplifications	Pages 7
Outcomes and prioritization	13	List and define all outcomes for which data will be sought, including prioritization of main and additional outcomes, with rationale	Pages 6
Risk of bias in individual studies	14	Describe anticipated methods for assessing risk of bias of individual studies, including whether this will be done at the outcome or study level, or both; state how this information will be used in data synthesis	Page 8
Data synthesis	15a	Describe criteria under which study data will be quantitatively synthesised	Pages 8-9
	15b	If data are appropriate for quantitative synthesis, describe planned summary measures, methods of handling data and methods of combining data from studies, including any planned exploration ofc onsistency (such as I^2, Kendall's τ)	Pages 8-9
	15c	Describe any proposed additional analyses (such as sensitivity or subgroup analyses, meta-regression)	Pages 8-9
	15d	If quantitative synthesis is not appropriate, describe the type of summary planned	N/A
Meta-bias(es)	16	Specify any planned assessment of meta-bias(es) (such as publication bias across studies, selective reporting within studies)	Page 9
Confidence in cumulative evidence	17	Describe how the strength of the body of evidence will be assessed (such as GRADE)	Page 9

For peer review only

*** It is strongly recommended that this checklist be read in conjunction with the PRISMA-P Explanation and Elaboration (cite when available) for important clarification on the items. Amendments to a review protocol should be tracked and dated. The copyright for PRISMA-P (including checklist) is held by the PRISMA-P Group and is distributed under a Creative Commons Attribution Licence 4.0.**

From: Shamseer L, Moher D, Clarke M, Ghersi D, Liberati A, Petticrew M, Shekelle P, Stewart L, PRISMA-P Group. Preferred reporting items for systematic review and meta-analysis protocols (PRISMA-P) 2015: elaboration and explanation. BMJ. 2015;349(jan02 1):g7647.

For peer review only

BMJ Open

Health effects of desert dust and sand storms: a systematic review and meta-analysis protocol

Journal:	*BMJ Open*
Manuscript ID	bmjopen-2019-029876.R2
Article Type:	Protocol
Date Submitted by the Author:	21-Jun-2019
Complete List of Authors:	Tobias, Aurelio; Spanish National Research Council, Institute of Environmental Assessment and Water Research<br>Karanasiou, Angeliki; Spanish National Research Council, Institute of Environmental Assessment and Water Research<br>Amato, Fulvio; National Spanish Research Council, Institute of Environmental Assessment and Water Research<br>Roqué, Marta; Iberoamerican Cochrane Centre; Biomedical Research Institute Sant Pau (IIB Sant Pau), CIBER Epidemiology and Public Health (CIBERESP)<br>Querol, Xavier; National Spanish Research Council, Institute of Environmental Assessment and Water Research
**Primary Subject Heading**:	Occupational and environmental medicine
Secondary Subject Heading:	Global health, Public health, Epidemiology
Keywords:	EPIDEMIOLOGY, PUBLIC HEALTH, Health policy < HEALTH SERVICES ADMINISTRATION & MANAGEMENT

SCHOLARONE™
Manuscripts

Health effects of desert dust and sand storms: a systematic review and meta-analysis protocol

Aurelio Tobías[1], Angeliki Karanasiou[1], Fulvio Amato[1], Marta Roqué[2,3], Xavier Querol[1]

1. Institute of Environmental Assessment and Water Research (IDAEA), Spanish Council for Scientific Research (CSIC), Barcelona, Spain.
2. Iberoamerican Cochrane Centre, Barcelona, Spain
3. Biomedical Research Institute Sant Pau (IIB Sant Pau), CIBER Epidemiology and Public Health (CIBERESP), Barcelona, Spain.

Address for correspondence:

Dr Aurelio Tobías

Institute of Environmental Assessment and Water Research (IDAEA),

Spanish Council for Scientific Research (CSIC),

C/ Jordi Girona 18-26

08034 Barcelona, Spain.

e-mail: aurelio.tobias@idaea.csic.es

Telf.: +34 934006100

For peer review only

ABSTRACT

Introduction: Desert dust concentrations raise concerns about adverse effects on human health. During the last decade, special attention has been given to mineral dust particles from desert dust and sand storms. However, evidence from previous reviews reported inconclusive results on their health effects and the biological mechanism remains unclear. We aim to systematically synthesise evidence on the health effects of desert dust and sand storms accounting for the relevant desert dust patterns from source areas and emissions, transport and composition.

Methods an Analysis: We will conduct a systematic review that investigated the health effects of desert dust and sand storms in any population. The search will be performed for any eligible studies from previous reviews and selected electronic databases until 2018. Study selection and reporting will follow the Preferred Reporting Items for Systematic Reviews and Meta-Analyses guidelines. Data from individual studies will be extracted using a standardised data extraction form. Quality of the studies will be assessed using a risk of bias tool for environmental exposures developed by experts convened by the World Health Organisation (WHO). A Meta-analysis will be performed by calculating the appropriate effect measures of association for binary and continuous outcomes from individual studies. Subgroup analyses will be performed by geographical areas to account for desert dust patterns.

Ethics and Dissemination: No primary data will be collected. For this reason, no formal ethical approval is required. This systematic review will help to fill the research gaps in the knowledge of desert dust on human health. The results will be disseminated through a WHO peer-reviewed publication and a conference presentation.

PROSPERO registration number: CRD42018091809

For peer review only

For peer review only - http://bmjopen.bmj.com/site/about/guidelines.xhtml

Strengths and limitations of this study

- This systematic review will be the first one to retrieve and evaluate published studies on the health effects of desert dust following a standardised protocol for data collection and reporting of findings.
- A meta-analysis will be used to provide evidence of the quantitative estimates of the health effects of desert dust exposures, accounting for relevant desert dust patterns from source areas, emissions, transport and composition.
- The diversity of methods to identify dust exposure events and the extensive health effects attributed to desert dust exposures might lead to study designs with different methodological characteristics, which will be carefully considered.
- The results of this systematic review will provide evidence to fill the gap in the knowledge of the health effects of desert dust and may help to develop appropriate preventive measures for dust episodes.

For peer review only

INTRODUCTION

Background

Desert dust and sand storms play a significant role in different aspects of weather, climate and atmospheric chemistry and represent a severe hazard to the environment and human health (1,2). Desert dust has a significant impact on air quality, not only in areas close to the source points or regions but over areas even a few thousands of kilometres distant (3,4). The air quality influence of dust episodes is a complex issue. It can increase particulate matter ambient concentrations by supplying large loads of mineral (or crustal) dust, but it can also carry anthropogenic pollutants, previously deposited in the source areas or trapped by the high dust air mass during its atmospheric transport (5,6), and carry large amounts of microorganisms and toxic biogenic allergens (7,8).

Research on the health effects of desert dust has increased substantially over the last decade. Although epidemiological studies from affected areas suggest the potential health effects of desert dust, the evidence remains unclear, as well as the biological mechanism. Previously published reviews, systematic or not, reported inconsistent results across studies and geographical regions (9-12). However, the reviewed studies differed in terms of settings, dust exposure assessment methods, outcomes and lagged exposures examined, and epidemiological study designs evaluated. For this reason, none of the previous reviews could perform a meta-analysis to summarize the health effects of desert dust quantitatively. Thus, a systematic review with a proper standardized protocol is required to provide a better knowledge of health effects and mechanism of toxicity of desert dust.

Objective

The objective of our systematic review is to summarize the evidence on the health effects of desert dust and sand storms, properly accounting for the relevant desert dust patterns from source areas and emissions, transport and composition. This systematic review will be carried out in the framework of the World Health Organisation (WHO) to inform the update of the WHO Global Air Quality Guidelines (AQGs), coordinated by the WHO Regional Office's European Centre for Environment and Health.

For peer review only - http://bmjopen.bmj.com/site/about/guidelines.xhtml

METHODS

This protocol has been registered in PROSPERO (CRD42018091809) and follows the Preferred Reporting Items for Systematic Review and Meta-Analysis Protocols (PRISMA-P) checklist (13) (online supplementary file 1). Any amendments to the protocol will be tracked and dated in PROSPERO.

Patient and public involvement

Patients and or public will not be involved in this study, as this is a systematic review protocol.

Study eligibility criteria

The WHO established the research question for this systematic review at the 3rd Meeting of the Global Platform on Air Quality and Health (Madrid, Spain, March 2017).

Population

This systematic review will include any human population, from developed and developing countries, living in urban and in rural areas exposed to desert dust and sand storms. Vulnerable population subgroups to the effects of desert dust and sand storms will be included, such as these with specific pre-existing health conditions (e.g. respiratory or cardiovascular diseases), pregnant women, newborns, children or the elderly. Whenever applicable, the considered health effect of exposure to desert dust in these vulnerable sub-groups of the population will be assessed separately.

Types of exposures

We will consider short and long-term exposure to desert dust characterized by source apportionment, component analysis, particle size and composition, use of weather data and back trajectories, or any other suitable method. The exposure to desert dust should be

For peer review only

compared with non-exposure to desert dust or exposure to lower concentrations of desert dust.

Types of outcome measures

We will consider any measurable independent change in the risk of adverse health effects (incidence or prevalence) related to exposure to desert dust as mortality for all-natural causes and cause-specific (e.g., cardiovascular and respiratory) and hospital admissions, emergency room admissions and emergency department specific causes. Moreover, based on a previous evaluation of the human health disorders related to desert dust (14), we will also consider cardiovascular and respiratory symptoms; lung diseases; coccidiomycosis; meningococcal meningitis; dermatological disorder; and deaths or injuries resulting from transport accidents.

Types of studies

This systematic review will consider all types of relevant studies published in peer-reviewed journals that assess the health effects of desert dust and sand storms. However, the studies will be classified according to their observational or experimental design (e.g., quasi-experimental and randomised controlled trials). The observational studies will also be classified at the ecological or individual level (e.g., surveys, case-control, cohort studies).

Information sources

We will systematically conduct a comprehensive literature search. Firstly, we will identify all studies already reported in previous reviews, systematic or not, on the health effects of desert dust and sand storms (9-12). Next, we will conduct a literature search for studies matching the study eligibility criteria in various databases, including MEDLINE (using PubMed) and EMBASE. Finally, references of identified relevant studies will be scanned to identify additional published data matching the study eligibility criteria. The search strategy has been developed in collaboration with a health sciences librarian specialising in systematic search procedures. We used free-text for each search engine, considering different definitions and keywords for desert dust and sand storms and health outcomes

For peer review only - http://bmjopen.bmj.com/site/about/guidelines.xhtml

(online supplementary file 2). We will retrieve eligible published studies until 2018 without language restrictions and will translate the non-English abstracts and studies, if necessary.

Selection of studies

Two reviewers will screen the titles and abstracts independently, record and import search results of their findings in EndNote X7 (Clarivate Analytics). All studies will be classified for inclusion or exclusion based on the eligibility of this systematic review and further checked for duplication. Full-text articles will be retrieved if the study is unclear from the title and abstract, and the relevant studies will be further confirmed for inclusion through full-text review. The third reviewer will resolve discrepancies between reviewers.

Data extraction

Reviewers will use a standardised data extraction form using Excel spreadsheet (Microsoft Corporation). Here, if a given study reports several health outcomes of interest, each outcome will take one record. Likewise, if a given study reports age or gender-specific effects, these will also be collected. Moreover, if a given study reports more than one effect estimate for each health outcome (e.g., for several lags), the one that the authors favoured most will be extracted. Main conclusion/findings for each study will also be collected as free text. The following characteristics of the included studies will be extracted:

- Citation details (e.g., first author, title, journal and date of publication).
- Study location (e.g. city, country, region), study period, characteristics of the study population (e.g., age, gender).
- Study design and statistical analysis.
- Method to identify dust events and exposure levels for days with and without dust events.
- Outcome assessment (e.g., cause of mortality, morbidity and other cause-specific outcomes).

For peer review only - http://bmjopen.bmj.com/site/about/guidelines.xhtml

- Confounders measured and adjusted for (e.g., meteorological variables and other air pollutants).
- Effect measures of association, and its 95% confidence interval, for days with and without dust events.

Quality assessment

We will use a new domain-based risk of bias tool to assess the quality of the evidence across studies for associations between specific air-pollutants and adverse health outcomes. The risk of bias tool, currently under development by a group of experts on environmental exposures convened by the WHO Guideline Development Group (GDG) for the AQGs, will adapt the domains according to the needs of the observational studies of exposure that form the body of the evidence in air-pollution. It will be revised and adapted to by the systematic review team members, and a member of the WHO GDG will support the application of the tool. Two reviewers will assess the risk of bias independently, and disagreements will be resolved through discussion.

Data synthesis and statistical analysis

For binary outcomes (e.g., mortality and morbidity), the relative risk (RR) will be used as the standard effect measure of association across studies. The hazard ratio (HR) if reported, may be considered equivalent to RRs. If Odds Ratios (OR) are reported in a given study and the outcome prevalence is higher than 10% they will be approximated as RRs (15) and similarly if the study design allows for it (e.g., time-series and time-stratified case-crossover (16)). Meta-analysis input data will be as the percentage increase in the RR (e.g., as %RR = (RR-1) x100%). When reporting effects of particulate matter for days with and without dust events the %RR will be standardised for a given increment of 10 $\mu g/m^3$, assuming a linear exposure-outcome relationship. For continuous outcomes, effect measures such as weighted mean difference (WMD) or standardized mean difference (SMD) may be used. The scale of the available data will be primarily used to determine the choice of effect measure if studies report effect estimates with the same or similar scale (WMD) or when the outcome is measured using different scales (SMD) (15).

For peer review only - http://bmjopen.bmj.com/site/about/guidelines.xhtml

The measures of the association from individual studies will be summarized in a meta-analysis estimate, with its 95% confidence interval, based on fixed or random effects model, depending on the heterogeneity of the analysis (15,17). Statistical heterogeneity of the effect estimates between studies included in the meta-analysis will be assessed using the I^2 statistic, where values of 25%, 50% and 75% will be considered as a low, moderate and high degree of heterogeneity, respectively (18). We will also report the between-study variance (τ^2) and the Cochran Q test for heterogeneity (19). All analyses will be done using Stata Statistical Software, release 15 (Stata Corp LP)

Due to the relevance of the study setting and dust exposure assessment, subgroup analyses will be done by geographical areas, considering studies conducted in Eastern Asia, Europe, Middle the East and other regions (e.g., Australia and North America). Subgroup analyses by pre-existing health conditions, age groups (14, 15-64, ≥65 years) and gender (male and female) will also be considered whenever data become available in the individual studies.

Assessment of reporting biases

Publication bias will be assessed when at least ten studies are included in the meta-analysis, using Begg's rank correlation test and examined using funnel plots (20).

Reporting conclusions

The systematic review will be conducted and reported in accordance to the Preferred Reporting Items for Systematic Review and Meta-Analysis (PRISMA) (21) standards, with slight adaptations since these were originally intended for health care intervention evaluation. We will base our conclusions only on findings from the quantitative or narrative synthesis of included studies for this systematic review. The quality of the evidence will be assessed using an adaptation for environmental studies, convened by the GDG for the AQGs, of the Grading of Recommendations Assessment (GRADE) (22). Studies that undergo meta-analyses will undergo GRADE assessment, but there might also other studies included

For peer review only

in the systematic review that could not be used in the meta-analysis but will be used for developing conclusions. The conclusions from this systematic review will be used to inform the update of the WHO AQGs, coordinated by the WHO Regional Office's European Centre for Environment and Health.

Ethics and dissemination

Formal ethical approval is not required, as no primary data will be collected. The results of this systematic review will fill the current research gaps in the knowledge on the health effects of desert dust and sand storms and would also help to alert the most sensitive population taking appropriate measures during dust episodes. All findings will be shared and disseminated by WHO in a peer-reviewed publication and conference presentation.

Acknowledgments

Ivan Solà (Iberoamerican Cochrane Centre) for their guidance in reviewing the search strategy proposed in this protocol.

Contributors: AT is the guarantor. AT drafted the protocol and registered in PROSPERO. MR and XQ reviewed and commented on the protocol in PROSPERO. AK, FA, MR and XQ all reviewed and commented on this protocol.

Funding: The World Health Organization, supported by the Ministry of Foreign Affairs of Norway, has funded this systematic review.

Competing interests: None declared.

Provenance and peer review: Commissioned by WHO; externally peer reviewed.

For peer review only

References

1. Mahowald NM, Kloster S, Engelstaedter S, Moore JK, Mukhopadhyay S, McConnell JR, et al. Observed 20th-century desert dust variability: impact on climate and biogeochemistry. Atmospheric Chemistry and Physics. 2010;10(22):10875-93.
2. De Longueville F, Hountondji YC, Henry S, Ozer P. What do we know about the effects of desert dust on air quality and human health in West Africa compared to other regions? Sci Total Environ. 2010;409(1):1-8.
3. Prospero JM. Environmental characterization of global sources of atmospheric soil dust identified with the NIMBUS 7 Total Ozone Mapping Spectrometer (TOMS) absorbing aerosol product. Reviews of Geophysics. 2002;40(1):1002.
4. Ginoux P, Prospero JM, Gill TE, Hsu NC, Zhao M. Global-scale attribution of anthropogenic and natural dust sources and their emission rates based on MODIS Deep Blue aerosol products. Reviews of Geophysics. 2012;50(3):1-36.
5. Mori I. Change in size distribution and chemical composition of Kosa (Asian dust) aerosol during long-range transport. Atmospheric Environment. 2003;37(30):4253-63.
6. Rodríguez S, Alastuey A, Alonso-Pérez S, Querol X, Cuevas E, Abreu-Afonso J, et al. Transport of desert dust mixed with North African industrial pollutants in the subtropical Saharan Air Layer. Atmospheric Chemistry and Physics. 2011;11(13):6663-85.
7. Ho H-M, Rao CY, Hsu H-H, Chiu Y-H, Liu C-M, Chao HJ. Characteristics and determinants of ambient fungal spores in Hualien, Taiwan. Atmospheric Environment. 2005;39(32):5839-50.
8. Griffin DW, Garrison VH, Herman JR, Shinn EA. African desert dust in the Caribbean atmosphere: Microbiology and public health. Aerobiologia. 2001;17(3):203-13.
9. Hashizume M, Ueda K, Nishiwaki Y, Michikawa T, Onozuka D. Health effects of Asian dust events: a review of the literature. Nihon Eiseigaku Zasshi. 2010;65(3):413-21.
10. Karanasiou A, Moreno N, Moreno T, Viana M, de Leeuw F, Querol X. Health effects from Sahara dust episodes in Europe: Literature review and research gaps. Environment International. 2012;47:107-14.

For peer review only

For peer review only - http://bmjopen.bmj.com/site/about/guidelines.xhtml

11. De Longueville F, Ozer P, Doumbia S, Henry S. Desert dust impacts on human health: an alarming worldwide reality and a need for studies in West Africa. Int J Biometeorol. 2013;57(1):1-19.

12. Zhang X, Zhao L, Tong D, Wu G, Dan M, Teng B. A Systematic Review of Global Desert Dust and Associated Human Health Effects. Atmosphere. 2016;7(12).

13. Shamseer L, Moher D, Clarke M, Ghersi D, Liberati A, Petticrew M, et al. Preferred reporting items for systematic review and meta-analysis protocols (PRISMA-P) 2015: elaboration and explanation. BMJ. 2015;349(jan02 1):g7647-g.

14. Goudie AS. Desert dust and human health disorders. Environment International. 2014;63:101-13.

15. Higgins JPT, Green S (editors). Cochrane handbook for systematic reviews of interventions version 5.1.0 [updated March 2011]. The Cochrane Collaboration; 2011. Available from http://www.handbook.cochrane.org. (accessed 15.02.2019).

16. Lu Y, Zeger SL. On the equivalence of case-crossover and time series methods in environmental epidemiology. Biostatistics. 2007;8(2): 337-44.

17. Borenstein M, Hedges LV, Higgins JPT, Rothstein HR. A basic introduction to fixed-effect and random-effects models for meta-analysis. Research Synthesis Methods. 2010;1(2):97-111.

18. Higgins JP, Thompson SG. Quantifying heterogeneity in a meta-analysis. Stat Med. 2002;21(11):1539-58.

19. Cochran WG. The χ test of goodness of fit. Annals of Mathematical Statistics 1952;23:315-45.

20. Begg CB, Mazumdar M. Operating characteristics of a rank correlation test for publication bias. Biometrics. 1994;50(2):1088-101.

21. Moher D, Liberati A, Tetzlaff J, Altman DG. Preferred reporting items for systematic reviews and meta-analyses: the PRISMA statement. BMJ. 2009;339(jul21 1):b2535-b.

22. Guyatt GH, Oxman AD, Vist GE, et al. GRADE Working Group. GRADE: an emerging consensus on rating quality of evidence and strength of recommendations. BMJ. 2008;336:924-6.

For peer review only - http://bmjopen.bmj.com/site/about/guidelines.xhtml

PRISMA-P (Preferred Reporting Items for Systematic review and Meta-Analysis Protocols) 2015 checklist: recommended items to address in a systematic review protocol*

**Section and topic**	**Item No.**	**Checklist item**	
**ADMINISTRATIVE INFORMATION**			
Title:			
Identification	1a	Identify the report as a protocol of a systematic review	Page 1
Update	1b	If the protocol is for an update of a previous systematic review, identify as such	N/A
Registration	2	If registered, provide the name of the registry (such as PROSPERO) and registration number	Pages 2-5
Authors:			
Contact	3a	Provide name, institutional affiliation, e-mail address of all protocol authors; provide physical mailing address of corresponding author	Page 1
Contributions	3b	Describe contributions of protocol authors and identify the guarantor of the review	Page 10
Amendments	4	If the protocol represents an amendment of a previously completed or published protocol, identify as such and list changes; otherwise, state plan for documenting important protocol amendments	Page 5
Support:			
Sources	5a	Indicate sources of financial or other support for the review	Page 10
Sponsor	5b	Provide name for the review funder and/or sponsor	Page 10

For peer review only

Role of sponsor or funder	5c	Describe roles of funder(s), sponsor(s), and/or institution(s), if any, in developing the protocol	Page 10
INTRODUCTION			
Rationale	6	Describe the rationale for the review in the context of what is already known	Page 4
Objectives	7	Provide an explicit statement of the question(s) the review will address with reference to participants, interventions, comparators, and outcomes (PICO)	Page 4-6
METHODS			
Eligibility criteria	8	Specify the study characteristics (such as PICO, study design, setting, time frame) and report characteristics (such as years considered, language, publication status) to be used as criteria for eligibility for the review	Pages 5-6
Information sources	9	Describe all intended information sources (such as electronic databases, contact with study authors, trial registers or other grey literature sources) with planned dates of coverage	Page 6
Search strategy	10	Present draft of search strategy to be used for at least one electronic database, including planned limits, such that it could be repeated	Page 6
Study records:			
Data management	11a	Describe the mechanism(s) that will be used to manage records and data throughout the review	Page 7
Selection process	11b	State the process that will be used for selecting studies (such as two independent reviewers) through each phase of the review (that is, screening, eligibility and inclusion in meta-analysis)	Pages 7-8

For peer review only

Data collection process	11c	Describe planned method of extracting data from reports (such as piloting forms, done independently, in duplicate), any processes for obtaining and confirming data from investigators	Page 7
Data items	12	List and define all variables for which data will be sought (such as PICO items, funding sources), any pre-planned data assumptions and simplifications	Pages 7
Outcomes and prioritization	13	List and define all outcomes for which data will be sought, including prioritization of main and additional outcomes, with rationale	Pages 6
Risk of bias in individual studies	14	Describe anticipated methods for assessing risk of bias of individual studies, including whether this will be done at the outcome or study level, or both; state how this information will be used in data synthesis	Page 8
Data synthesis	15a	Describe criteria under which study data will be quantitatively synthesised	Pages 8-9
	15b	If data are appropriate for quantitative synthesis, describe planned summary measures, methods of handling data and methods of combining data from studies, including any planned exploration ofc onsistency (such as I^2, Kendall's τ)	Pages 8-9
	15c	Describe any proposed additional analyses (such as sensitivity or subgroup analyses, meta-regression)	Pages 8-9
	15d	If quantitative synthesis is not appropriate, describe the type of summary planned	N/A
Meta-bias(es)	16	Specify any planned assessment of meta-bias(es) (such as publication bias across studies, selective reporting within studies)	Page 9
Confidence in cumulative evidence	17	Describe how the strength of the body of evidence will be assessed (such as GRADE)	Page 9

For peer review only

*** It is strongly recommended that this checklist be read in conjunction with the PRISMA-P Explanation and Elaboration (cite when available) for important clarification on the items. Amendments to a review protocol should be tracked and dated. The copyright for PRISMA-P (including checklist) is held by the PRISMA-P Group and is distributed under a Creative Commons Attribution Licence 4.0.**

From: Shamseer L, Moher D, Clarke M, Ghersi D, Liberati A, Petticrew M, Shekelle P, Stewart L, PRISMA-P Group. Preferred reporting items for systematic review and meta-analysis protocols (PRISMA-P) 2015: elaboration and explanation. BMJ. 2015;349(jan02 1):g7647.

For peer review only

Supplementary File 2.

PubMed search strategy:

(Desert dust[Title/Abstract] OR Sand storms[Title/Abstract] OR Dust episode[Title/Abstract] OR Dust outbreaks[Title/Abstract] OR Asian dust[Title/Abstract] OR Saharan dust[Title/Abstract] OR Yellow dust[Title/Abstract] OR Kosa[Title/Abstract] OR Calima[Title/Abstract]) AND ((mortality[Title/Abstract] OR death[Title/Abstract]) OR (morbidity[Title/Abstract] OR hospital admissions[Title/Abstract] OR emergency admissions[Title/Abstract] OR emergency room[Title/Abstract] OR hospital clinic[Title/Abstract] OR visits[Title/Abstract] OR calls[Title/Abstract]) OR (cardiovascular[Title/Abstract] OR cardicac[Title/Abstract] OR respiratory[Title/Abstract] OR infectious[Title/Abstract] OR coccidiomycosis[Title/Abstract] OR meningitis[Title/Abstract] OR dermatological [Title/Abstract] OR accidents[Title/Abstract])))

EMBASE search strategy:

('Desert dust':ab,ti OR 'Sand storms':ab,ti OR 'Dust episode':ab,ti OR 'Dust outbreaks':ab,ti OR 'Asian dust':ab,ti OR 'Saharan dust':ab,ti OR 'Yellow dust':ab,ti OR 'Kosa':ab,ti OR 'Calima':ab,ti) AND (('mortality':ab,ti OR 'death':ab,ti) OR ('morbidity':ab,ti OR 'hospital admissions':ab,ti OR 'emergency admissions':ab,ti OR 'emergency room':ab,ti OR 'clinic':ab,ti OR 'visits':ab,ti OR 'calls':ab,ti) OR ('cardiovascular':ab,ti OR 'cardicac':ab,ti OR 'respiratory':ab,ti OR 'infectious':ab,ti OR 'coccidiomycosis':ab,ti OR 'meningitis':ab,ti OR 'dermatological':ab,ti OR 'accidents':ab,ti)))

For peer review only
